# Roles of RNA-binding proteins in macrophage function regulation and immunotherapy

**DOI:** 10.3389/fcell.2026.1771892

**Published:** 2026-02-23

**Authors:** Jia Chen, Xiulin Jiang, Yixiao Yuan, Chunhong Li, Chongxin Li, Qiang Zhou, Qiang Wang, Weiwei Bai

**Affiliations:** 1 Department of urology, Hebei Petro China Central Hospital, Langfang, China; 2 Department of Systems Biology, City of Hope Comprehensive Cancer Center, Biomedical Research Center, Monrovia, CA, United States; 3 Department of Oncology, Suining Central Hospital, Suining, Sichuan, China; 4 Department of Oncology, Qujing Central Hospital of Yunnan Province, Qujing, Yunan, China; 5 Department of Gastrointestinal Surgical Unit, Suining Central Hospital, Suining, Sichuan, China

**Keywords:** cell survival, inflammation, macrophage activation, post-transcriptional regulation, programmed cell death, RBPs

## Abstract

Macrophages are essential components of the innate immune system and exhibit remarkable functional plasticity, playing pivotal roles in inflammatory responses, maintenance of tissue homeostasis, and the initiation and progression of tumors as well as a wide range of other diseases. Accumulating evidence in recent years has demonstrated that, in addition to classical transcriptional regulation, post-transcriptional regulation is equally critical for macrophage fate determination and functional specialization. RNA-binding proteins (RBPs), as central regulators of post-transcriptional gene control, orchestrate a sophisticated and dynamic gene expression network by modulating RNA splicing, nucleocytoplasmic transport, stability and decay, translational efficiency, RNA epigenetic modifications, liquid–liquid phase separation, and chromatin-associated processes. Substantial experimental data indicate that RBPs are deeply involved in macrophage polarization, survival and programmed cell death, as well as metabolic reprogramming, thereby shaping the magnitude of inflammatory responses, immune suppressive states, and remodeling of the tumor microenvironment. In this review, we systematically summarize the molecular mechanisms by which RBPs regulate macrophage functions, with particular emphasis on their roles in inflammatory disorders, cancer, and metabolism-related diseases. We also highlight recent advances in the coordinated regulation of macrophage biology by RBPs in conjunction with RNA modifications, including m^6^A, m^5^C, and ac^4^C, as well as noncoding RNAs. Finally, we discuss the opportunities and challenges of targeting RBPs as emerging immunotherapeutic strategies, underscoring their potential in reprogramming the tumor immune microenvironment and enhancing the efficacy of immunotherapy, thereby providing a theoretical framework for the development of precise immune intervention approaches.

## 1 Introduction

Macrophages are essential components of the innate immune system and play central roles in maintaining immune homeostasis, regulating inflammatory responses, and driving the initiation and progression of a wide range of diseases (Xia et al., 2020). Beyond their classical functions in pathogen clearance and antigen presentation, macrophages exhibit remarkable functional plasticity in response to microenvironmental cues (Xia et al., 2020). In settings such as infection, tissue injury, and the tumor microenvironment, macrophages can adopt distinct activation states that either amplify inflammation or promote immunosuppression, thereby profoundly influencing disease progression and therapeutic outcomes (Yu et al., 2023). Consequently, elucidating the molecular mechanisms that govern macrophage functional regulation is of fundamental importance for understanding immune response modulation and for developing novel therapeutic strategies (Yu et al., 2023). Recent studies have revealed that immune cell fate determination is not solely dictated by transcriptional regulation; post-transcriptional regulation is equally indispensable in shaping immune cell identity and function (Yu et al., 2023). Processes such as mRNA splicing, stability, subcellular localization, and translational efficiency enable rapid and fine-tuned modulation of protein expression programs, allowing immune cells to respond swiftly to dynamic and complex microenvironments (Li et al., 2023). Compared with transcriptional control, post-transcriptional regulation is characterized by faster kinetics and greater reversibility, making it particularly well suited for regulating the dynamic functional states of immune cells (Li et al., 2023). Post-transcriptional regulation, including RBPs and RNA modifications, plays a crucial role in controlling gene expression in immune cells, particularly macrophages. Macrophages are highly plastic and can adopt diverse functional states in response to environmental cues, which is critical in the context of various diseases such as chronic inflammation, cancer, and atherosclerosis. Dysregulation of RBPs or RNA modifications can alter macrophage polarization and function, thereby influencing disease progression (Fernandez et al., 2022). Understanding these regulatory mechanisms not only provides fundamental insights into macrophage biology but also highlights potential therapeutic strategies, including targeted modulation of RBPs or RNA modifications, for treating inflammatory and immune-related diseases (Teixeira-Coelho et al., 2014).

RBPs are central mediators of post-transcriptional gene regulation. By recognizing and binding specific RNA sequences or structural motifs, RBPs regulate RNA splicing, modification, stability, transport, and translation (Finan et al., 2023). The functional specificity of RBPs is determined by their RNA-binding domains (RBDs), which confer both affinity and selectivity for RNA targets (Finan et al., 2023). To date, more than 30 distinct types of RBDs have been identified, among which RNA recognition motifs (RRMs), KH domains, helicase domains, and intrinsically disordered regions (IDRs) are the most common (Qin et al., 2020). RRMs and KH domains typically mediate highly selective binding through recognition of specific nucleotide sequences, whereas helicase domains bind RNA backbones in an ATP-dependent manner to remodel RNA secondary structures (Qin et al., 2020). In contrast, IDRs lack a fixed three-dimensional structure but enhance binding versatility through flexible, multivalent interactions. The combinatorial and cooperative actions of distinct RBDs enable RBPs to precisely control RNA processing, stability, translation, and subcellular localization, thereby exerting broad regulatory influence over gene expression networks (Qin et al., 2020).

Recent studies have highlighted the dynamic interplay between tumor cells and the immune microenvironment as a critical determinant of cancer progression and therapeutic response (Ke et al., 2025; Zhang et al., 2025). For example, iMRS enables personalized prediction of immunotherapy response and prognosis in LUAD, where high iMRS—driven by the immune-exclusion–related oncogene SLC25A1—defines immune-cold tumors with poor outcomes, while targeting SLC25A1 enhances CD8^+^ T cell–mediated immunity and improves anti–PD-1 efficacy (Zhang et al., 2025). In particular, macrophage-driven remodeling of the tumor microenvironment (TME) has emerged as a key mechanism underlying immunosuppressive states, metabolic adaptation, and resistance to immunotherapy (Gong et al., 2025). Growing evidence indicates that post-transcriptional regulatory programs, including those orchestrated by RBPs, are central to shaping macrophage phenotypes and their functional plasticity within the TME, thereby influencing antitumor immunity and treatment outcomes (Gong et al., 2025). EGFR-mutant LUAD exhibits an immunosuppressive tumor immune microenvironment characterized by TIGIT^+^ regulatory T cells, neutrophils, and macrophages, whereas EGFR wild-type tumors display an immune-active TIME with ZNF683^+^ CD8^+^ tissue-resident memory T cells, diverse memory B cells, and FGFBP2^+^ CD16^high^ NK cells, highlighting distinct immune landscapes that can guide precision immunotherapy (Gong et al., 2025).

Through the assembly of intricate RNA–protein regulatory networks, RBPs serve as pivotal hubs in maintaining cellular homeostasis and coordinating stress responses (Qin et al., 2020). Increasing evidence highlights the critical roles of RBPs in immune cells, particularly macrophages. In macrophages, RBPs regulate the expression of inflammatory mediators, growth factors, and metabolic genes, thereby influencing macrophage polarization, survival and programmed cell death, as well as metabolic reprogramming (Chen et al., 2021). These regulatory processes determine macrophage functional orientation in both acute and chronic inflammation and play crucial roles in cancer, infectious diseases, and metabolic disorders. However, a systematic understanding of RBPs in macrophage biology remains limited, and their integrated roles within immune regulatory networks have not yet been fully elucidated. Therefore, this review aims to comprehensively summarize the roles of RBPs in regulating macrophage polarization, survival and programmed cell death, metabolic reprogramming, and disease progression, with a particular focus on their functional significance in tumor immunity and inflammatory diseases. Furthermore, we explore the potential of RBPs as emerging immunotherapeutic targets. By integrating current research advances, this review seeks to provide a theoretical framework for understanding post-transcriptional regulation in macrophages and for developing novel immunotherapeutic strategies.

## 2 Functions of RBPs

**FIGURE 1:**
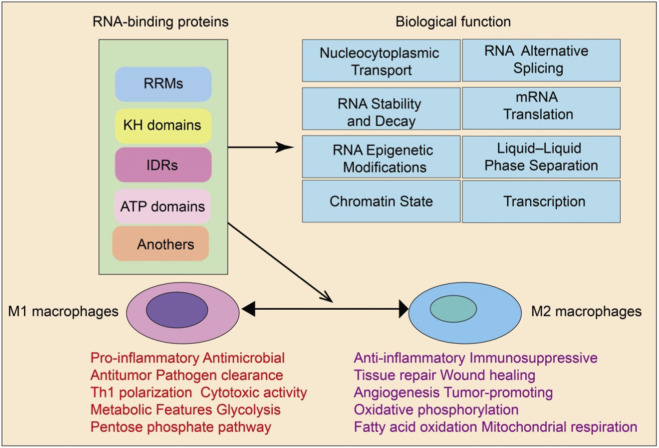
Functional roles of RBPs in macrophage polarization. RBPs containing RRMs, KH domains, IDRs, ATP-binding domains, and other motifs regulate multiple biological functions, including nucleocytoplasmic transport, RNA alternative splicing, mRNA stability and decay, translation, RNA epigenetic modifications, liquid–liquid phase separation, chromatin state, and transcription. These RBPs contribute to the polarization of macrophages into M1 (pro-inflammatory, antimicrobial, antitumor) or M2 (anti-inflammatory, immunosuppressive, tissue repair) phenotypes, with distinct metabolic programs such as glycolysis versus oxidative phosphorylation.

Collectively, RBPs orchestrate a highly dynamic and finely tuned gene expression regulatory network by acting across multiple layers, including transcription, RNA splicing, nucleocytoplasmic transport, RNA stability and decay, translational control, RNA epigenetic modification, liquid-liquid phase separation, and chromatin state regulation (Zhu W. et al., 2025). RBPs not only serve as core regulators of post-transcriptional processes but also intersect functionally with chromatin and transcriptional machinery, enabling integrated control over the entire gene expression continuum (Zhu W. et al., 2025). This multilayered and spatiotemporally specific regulatory capacity positions RBPs as critical molecular hubs linking transcriptional programs to protein output. Their functional plasticity provides the molecular basis for rapid cellular adaptation to environmental changes and offers important insights into disease mechanisms and therapeutic development (Figure 1).

### 2.1 Regulation of RNA nucleocytoplasmic transport by RBPs

The export of RNA from the nucleus to the cytoplasm is a prerequisite for its biological function (Tian and Manley, 2017). RBPs regulate nucleocytoplasmic RNA distribution by recognizing specific RNA sequences or structures and mediating interactions between RNA, nuclear pore complexes, and transport factors.For example, YTHDC1 is a nuclear m^6^A reader protein that plays a pivotal role in RNA nucleocytoplasmic transport (Widagdo et al., 2022). As a primary nuclear sensor of m^6^A-modified transcripts, YTHDC1 selectively binds m^6^A-modified mRNAs or long noncoding RNAs and functions as a molecular adaptor to facilitate their interaction with nuclear export machinery (Widagdo et al., 2022). Mechanistically, YTHDC1 recruits nuclear export adaptors such as SRSF3 and promotes their coupling to nuclear pore–associated transport pathways, thereby enhancing the export of specific RNAs from the nucleus to the cytoplasm (Widagdo et al., 2022). Concurrently, YTHDC1 antagonizes nuclear retention factors such as SRSF10, further fine-tuning the balance between nuclear retention and cytoplasmic export. This selective transport mechanism ensures that functional transcripts are efficiently delivered to the cytoplasm for translation.Under both physiological and pathological conditions, YTHDC1-mediated RNA export influences cell differentiation, stress responses, and tumorigenesis (Widagdo et al., 2022). Dysregulation of this process can lead to aberrant nuclear accumulation or mislocalization of regulatory RNAs, resulting in widespread perturbation of gene expression programs. Overall, by coupling m^6^A recognition to RNA export pathways, YTHDC1 occupies a central position in post-transcriptional regulatory networks and provides a paradigm for understanding the functional interplay between RNA modification and nucleocytoplasmic transport. Through such mechanisms, RBPs not only define the spatial and temporal expression patterns of RNAs but also enable rapid cellular adaptation to environmental cues (Jiang et al., 2021).

### 2.2 Regulation of alternative RNA splicing by RBPs

Alternative splicing greatly expands the coding potential of the genome, and RNA-binding proteins (RBPs) serve as central regulators of exon selection and splice site usage. SRSF1 (also known as ASF/SF2) is a prototypical serine/arginine-rich splicing factor that plays a pivotal role in this process (Sokół et al., 2017). Mechanistically, SRSF1 binds to exon splicing enhancers (ESEs) via its RNA recognition motifs, promoting spliceosome assembly by stabilizing the association of U1 snRNP with 5′splice sites and U2AF with 3′splice sites. Depending on the local RNA sequence context and its interactions with other splicing regulators, SRSF1 can either enhance exon inclusion or repress exon usage, fine-tuning splicing outcomes. In addition, the phosphorylation status of SRSF1 and its dynamic nuclear localization critically influence its activity. Through the regulation of alternative splicing, SRSF1 generates transcript isoforms with distinct or even opposing functions, which can profoundly impact cell fate and disease progression. In macrophages, alternative splicing regulated by SRSF1 may modulate the expression of key cytokines or signaling molecules, thereby shaping polarization states and immune responses (Sokół et al., 2017).

### 2.3 Regulation of RNA stability and decay by RBPs

RNA stability directly determines transcript abundance and functional duration within the cell. RBPs regulate RNA turnover by binding specific RNA regions and modulating interactions with degradation or protective machineries.IGF2BP2 is a representative m^6^A-dependent RNA stabilizing factor that enhances transcript stability by recognizing m^6^A-modified sites on target mRNAs (Liu S. et al., 2025). Mechanistically, IGF2BP2 prevents the recruitment of RNA decay complexes or recruits stabilizing cofactors, thereby prolonging mRNA half-life. This protective mechanism sustains expression of target genes involved in metabolism, cell proliferation, and tumorigenesis (Liu S. et al., 2025). In contrast, YTHDF2 functions as a key m^6^A-dependent RNA decay factor. By recognizing m^6^A-modified transcripts, YTHDF2 directs them to RNA decay pathways, promoting deadenylation, decapping, and subsequent degradation (Chen et al., 2022). Together, stabilizing factors such as IGF2BP2 and degradative factors such as YTHDF2 establish a dynamic balance that governs RNA fate and maintains gene expression homeostasis.

### 2.4 Regulation of mRNA translation by RBPs

mRNA translation constitutes the critical link between gene expression and protein function. RBPs directly modulate translational output by influencing ribosome recruitment, translation initiation, or elongation.

YTHDF1 is a cytoplasmic m^6^A reader protein that enhances translational efficiency by coupling m^6^A-modified mRNAs to the translational machinery (Ren et al., 2023). By recognizing m^6^A sites on target transcripts, YTHDF1 promotes ribosome recruitment and assembly, thereby facilitating translation initiation (Ren et al., 2023). This mechanism enables preferential and efficient translation of modified mRNAs. Dysregulation of YTHDF1-mediated translation affects cellular stress responses, differentiation, and tumorigenesis, underscoring its importance in maintaining proteomic balance (Li et al., 2025a). Through translational control, RBPs allow cells to rapidly adjust protein expression without altering mRNA abundance.

### 2.5 Regulation of RNA epigenetic modifications by RBPs

RNA epigenetic modifications provide a sophisticated layer of post-transcriptional control. RBPs are central to this process, acting either as “writers” that deposit modifications or “readers” that interpret them to dictate RNA fate (Li et al., 2025b). RBPs can function as readers of RNA modifications or influence the activity of RNA-modifying enzymes. For example, N6-methyladenosine (m6A): The most prevalent internal mRNA modification, m^6^A, is catalyzed by a multicomponent “writer” complex. METTL3 serves as the catalytic core, working in tandem with METTL14 and WTAP to deposit m^6^A marks primarily within coding regions and 3′untranslated regions (3′UTRs). These marks create specific docking sites for downstream reader proteins that influence mRNA splicing, export, and translation (Zeng et al., 2020). N4-acetylcytidine (ac4C): Beyond methylation, RBPs like NAT10 facilitate ac^4^C modification on mRNA and rRNA. Functioning as a primary acetyltransferase, NAT10 enhances RNA stability and ensures translational fidelity (Wang et al., 2025). Unlike transient signals, ac^4^C represents a relatively stable “gain-of-function” modification.By recognizing and modulating these epigenetic marks, RBPs integrate various cellular signals to precisely fine-tune RNA metabolism (Wang et al., 2024).

### 2.6 Regulation of liquid–liquid phase separation by RBPs

Liquid–liquid phase separation (LLPS) has emerged as a fundamental mechanism for the formation of membrane-less cellular compartments that organize RNA metabolism and gene regulation (Hyman et al., 2014). This process is typically driven by multivalent interactions among proteins and RNAs, allowing specific molecules to dynamically concentrate without the need for a surrounding membrane. RBPs are key drivers of LLPS because they often contain intrinsically disordered regions and multiple RNA-binding domains, enabling them to engage in numerous weak, reversible interactions (Hyman et al., 2014). In this context, RBPs can act as molecular scaffolds that nucleate and stabilize phase-separated condensates. Ecent studies have shown that the m^6^A reader YTHDC1 participates in RNA-dependent LLPS within the nucleus (Chen et al., 2025a). First, YTHDC1 recognizes m^6^A-modified RNA molecules through its YTH domain. Second, YTHDC1 interacts multivalently with other nuclear RBPs, splicing factors, and chromatin-associated regulators. These combined RNA–protein and protein–protein interactions promote the assembly of dynamic and reversible RNA–protein condensates enriched in specific transcripts (Cheng et al., 2021). By enhancing the scaffold function of m^6^A-modified RNAs, YTHDC1 increases local protein concentration and stabilizes phase-separated states. This compartmentalization enables the spatial segregation of RNA processing machinery, thereby facilitating efficient and coordinated post-transcriptional regulation. Together, these findings provide a mechanistic framework for understanding how RBPs and RNA modifications cooperatively regulate LLPS and contribute to the spatial organization of gene expression programs (Cheng et al., 2021).

### 2.7 Regulation of chromatin state by RBPs

Although traditionally regarded as post-transcriptional regulators, RBPs have increasingly been implicated in chromatin regulation through interactions with chromatin-associated RNAs or chromatin-modifying complexes (Chen et al., 2024). NSUN2 is an m^5^C methyltransferase best known for modifying mRNA, tRNA, and select noncoding RNAs. Beyond its canonical post-transcriptional roles (Li et al., 2025c), NSUN2 can indirectly influence chromatin states (Cheng et al., 2018). By modifying specific RNAs, NSUN2 regulates their interactions with chromatin-binding proteins or transcriptional regulators. m^5^C-modified RNAs may act as molecular scaffolds to recruit or stabilize chromatin regulatory complexes, thereby altering chromatin accessibility and transcriptional activity (Cheng et al., 2018). These findings extend the functional repertoire of RBPs and highlight their bridging role between transcriptional and post-transcriptional regulation.

### 2.8 Regulation of transcription by RBPs

In addition to post-transcriptional regulation, certain RBPs directly or indirectly participate in transcriptional control by modulating transcription complex assembly or RNA polymerase II activity. RBM25 is a representative RBP traditionally associated with alternative splicing regulation (Xiao et al., 2019). Emerging evidence indicates that RBM25 also influences transcription through RNA-dependent mechanisms. RBM25 binds specific pre-mRNAs or noncoding RNAs and modulates their interactions with transcriptional machinery, transcription factors, or chromatin regulators, thereby affecting transcription efficiency and start site selection. By altering the composition and localization of RNA–protein complexes at promoters or enhancers, RBM25 influences RNA polymerase II recruitment and elongation (Xiao et al., 2019). These findings further underscore the central role of RBPs in coordinating gene expression across multiple regulatory layers.

## 3 Regulatory mechanisms of RBPs in macrophage polarization

While the diverse functions of RNA-binding proteins have been broadly characterized, their cell type–specific regulatory roles in shaping macrophage phenotypes remain incompletely understood. Given that macrophage polarization represents a central determinant of immune responses and tissue homeostasis, the following section focuses on the molecular mechanisms by which RBPs orchestrate macrophage polarization through multilayered post-transcriptional regulation.

### 3.1 Molecular basis of macrophage polarization

Macrophages exhibit remarkable functional plasticity and can undergo profound phenotypic and functional reprogramming in response to diverse microenvironmental cues (Pidwill et al., 2020). The most extensively characterized polarization states are the classically activated pro-inflammatory (M1) phenotype and the alternatively activated anti-inflammatory or tissue-repairing (M2) phenotype (Pidwill et al., 2020). M1 macrophages are typically induced by stimuli such as lipopolysaccharide (LPS) and interferon-γ (IFN-γ) and are characterized by high expression of pro-inflammatory cytokines and antigen-presenting molecules, thereby playing critical roles in pathogen clearance and antitumor immunity (Pidwill et al., 2020). In contrast, M2 macrophages are driven by signals including interleukin-4 (IL-4) and interleukin-13 (IL-13) and primarily contribute to immune suppression, tissue remodeling, wound healing, and tumor progression (Korf et al., 2019). At the molecular level, macrophage polarization is orchestrated by the coordinated activation of multiple canonical signaling pathways. The NF-κB pathway serves as a central driver of M1 polarization by rapidly inducing the transcription of pro-inflammatory genes such as TNF-α, IL-6, and IL-1β. STAT1 and members of the interferon regulatory factor (IRF) family play indispensable roles in IFN-γ–mediated inflammatory amplification and antimicrobial responses (Korf et al., 2019). Conversely, STAT6 functions as a core transcription factor in M2 polarization, activating genes associated with tissue repair and immune suppression, including Arg1 and Mrc1. Together, these pathways constitute the transcriptional framework underlying macrophage polarization (Korf et al., 2019). However, increasing evidence indicates that transcriptional regulation alone is insufficient to explain the rapid, reversible, and highly fine-tuned phenotypic transitions observed during macrophage polarization. Post-transcriptional regulation, by modulating mRNA splicing, stability, degradation rates, and translational efficiency, provides an additional and essential regulatory layer (Castegna et al., 2020). Under inflammatory stimulation or microenvironmental changes, post-transcriptional mechanisms can swiftly remodel the proteomic landscape without requiring *de novo* transcription, enabling macrophages to rapidly switch functional states. RBPs act as key executors of this post-transcriptional regulatory network. By selectively binding mRNAs encoding inflammatory mediators, signaling molecules, and metabolic regulators, RBPs determine the fate of these transcripts under M1-or M2-polarizing conditions (Castegna et al., 2020). Through precise control of the magnitude and duration of effector molecule expression, RBPs play fundamental roles in maintaining polarization stability, fine-tuning polarization intensity, and mediating transitions between different macrophage states. Thus, elucidating macrophage polarization from a post-transcriptional perspective is critical for a comprehensive understanding of immune regulation and provides a conceptual foundation for dissecting RBP-mediated mechanisms.

### 3.2 The mechanism of RBPs in the polarization of macrophages

**FIGURE 2:**
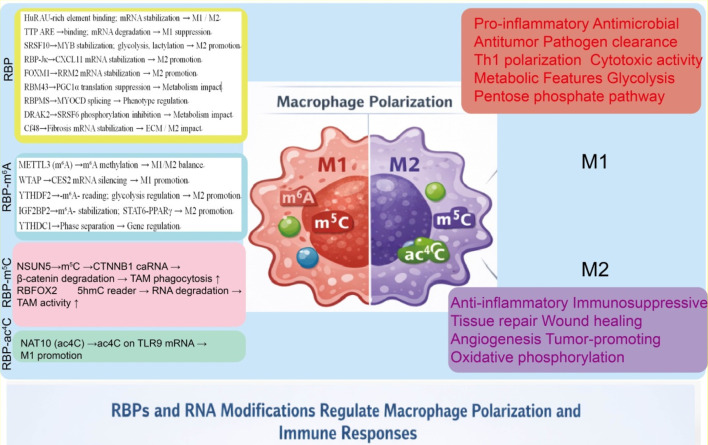
RBPs and RNA modifications regulate macrophage polarization and immune functions. RBPs and RNA modifications, including m^6^A, m^5^C and ac^4^C, coordinately regulate macrophage polarization and function. These regulatory layers influence M1/M2 polarization by modulating mRNA stability, translation, metabolic reprogramming, and inflammatory signaling. Notably, NSUN5-mediated m^5^C modification of CTNNB1 chromatin-associated RNA promotes β-catenin degradation via the TET2–RBFOX2 axis, enhancing tumor-associated macrophage (TAM) phagocytic activity and antitumor immunity.

Accumulating evidence indicates that RNA-binding proteins and RNA epigenetic modifications critically shape macrophage polarization and function, thereby remodeling the tumor immune microenvironment (Figure 2). In Table 1, we summarizes representative RNA-binding proteins and RNA modifications that regulate macrophage polarization and function by controlling RNA stability, translation, and metabolic programs.

**TABLE 1:** Key RNA-binding proteins and RNA modifications regulating macrophage polarization and immune functions.

RBP	Mechanism	Effect on macrophage polarization	Pathological context	Therapeutic potential	Ref
HuR (ELAVL1)	Binds AU-rich elements in 3′UTRs; stabilizes mRNAs	Promotes both M1 (pro-inflammatory cytokines) and M2 (IL-10) phenotypes depending on context	Autoimmune diseases (imbalanced immune suppression)	Targeting HuR could rebalance cytokine expression	Zhang et al. (2022a)
TTP	Binds AREs in TNF-α, IL-6 mRNAs; promotes degradation	Limits M1 pro-inflammatory responses	Chronic inflammatory diseases	Modulation may prevent excessive inflammation	Zhang et al. (2020)
SRSF10	Stabilizes MYB mRNA; promotes glycolysis and H3K18 lactylation	Indirectly promotes M2 polarization in tumor microenvironment	Tumor immunosuppressive microenvironment; immunotherapy resistance	Targeting SRSF10 may enhance immunotherapy efficacy	Luo et al. (2024)
METTL3 (m^6^A)	m^6^A methylation of SPRED2, other transcripts	Suppresses pro-tumorigenic polarization in TAMs; regulates M1/M2 balance	Cancer, ulcerative colitis	Pharmacological modulation can reshape TME and improve PD-1 therapy	Yin et al. (2021)
WTAP (with YTHDF2)	Silences CES2 mRNA via m^6^A	Promotes M1 polarization; CD4^+^ T cell infiltration	Ulcerative colitis	Inhibition alleviates disease severity	Xie et al. (2024)
YTHDF2	m6A reader; regulates glycolysis, IFN-γ response	Limits M1; promotes immunosuppressive M2 in tumors	Tumor immune evasion	Targeting YTHDF2 enhances immune-activated TME and checkpoint therapy	Yang et al. (2024)
IGF2BP2	m^6^A-dependent stabilization of TSC1; STAT6–HMGA2–PPARγ axis	Promotes M2 polarization; suppresses M1	Allergic inflammation, tumor progression	Inhibition shifts polarization toward M1; reduces immunosuppression	Wang et al. (2021)
NAT10 (ac^4^C)	Catalyzes ac^4^C on TLR9 mRNA	Promotes M1 polarization; enhances pro-inflammatory cytokine expression	Atherosclerosis	NAT10 inhibition promotes M2 transition and alleviates AS progression	Yin et al. (2025)
YTHDC1	RNA-dependent phase separation	Facilitates dynamic RNA-protein condensates influencing gene expression	General macrophage activation context	Modulating phase separation may adjust macrophage responses	Cheng et al. (2021)
RBP-Jκ	Stabilizes CXCL11 mRNA	Enhances metastatic TAM functions	Colorectal cancer	Targeting RBP-Jκ may reduce metastasis	Liu et al. (2021)
FOXM1/IGF2BP3	Stabilizes RRM2 mRNA via m^6^A	Promotes M2 polarization; suppresses ferroptosis	Hepatocellular carcinoma	Targeting axis enhances ferroptosis and anti-tumor immunity	Gao et al. (2025)
RBM43	Suppresses PGC1α mRNA translation	Impairs oxidative metabolism; affects macrophage energy balance	Obesity-associated metabolic diseases	Deletion restores metabolism; potential therapeutic target	Dumesic et al. (2025)
RBPMS	Modulates MYOCD splicing	Supports VSMC contractile phenotype; influences macrophage-related vascular remodeling	Atherosclerosis, restenosis	Potential target to prevent vascular remodeling	Imamura et al. (2010)
DRAK2	Inhibits SRSF6 phosphorylation; regulates mitochondrial gene splicing	Impacts macrophage metabolic function indirectly	NASH/NAFLD	Liver-specific targeting suppresses disease progression	Li et al. (2021)
Cf48	Stabilizes fibrosis-related mRNAs	Enhances fibrotic responses; macrophage-mediated ECM deposition	CKD	Inhibition attenuates renal fibrosis	Li et al. (2025b)

#### 3.2.1 The mechanism of RBPs in the polarization of M1 macrophages

RBPs regulate inflammatory gene expression through selective interactions with specific RNA sequences or structural motifs (Chen et al., 2020). Many mRNAs encoding pro-inflammatory and anti-inflammatory cytokines harbor AU-rich elements (AREs) within their untranslated regions, which serve as key binding sites for RBPs that control mRNA stability and degradation (Chen et al., 2020). For instance, tristetraprolin (TTP) binds to AREs in TNF-α and IL-6 mRNAs, promoting their degradation and thereby limiting excessive inflammatory responses (Shi et al., 2012). In contrast, HuR (ELAVL1) recognizes similar sequences but enhances mRNA stability and translational efficiency, leading to sustained cytokine production (Zhang et al., 2022a). This mode of regulation affects not only mRNA half-life but also translational output, allowing RBPs to dynamically adjust the expression of M1-or M2-associated genes in response to environmental stimuli (Ikegawa et al., 2024). Anti-inflammatory cytokines such as IL-10 are also subject to fine regulation by RBPs; HuR-mediated stabilization of IL-10 mRNA supports the maintenance of anti-inflammatory phenotypes, underscoring the pivotal role of RBPs in balancing inflammation and immune suppression (Ikegawa et al., 2024). Beyond classical ARE-mediated regulation, RBPs participate in complex metabolic–epigenetic–immune crosstalk. The splicing factor SRSF10 stabilizes MYB mRNA in tumor cells, thereby activating glycolysis, increasing lactate production, and inducing histone H3K18 lactylation (Cai et al., 2024).

RNA modifications such as m^6^A, m^5^C, and ac^4^C represent an additional layer of post-transcriptional regulation that can fine-tune gene expression in immune cells (Figure 2). By affecting RNA stability, splicing, localization, and translation, these modifications influence macrophage activation and polarization, thereby shaping immune responses under physiological and pathological conditions. Here, we summarize the current understanding of the roles of these RNA modifications in regulating macrophage function.

The m^6^A methyltransferase METTL3 exerts context-dependent effects in macrophages. In tumor-associated macrophages, METTL3 suppresses pro-tumorigenic polarization through YTHDF1-dependent translational control of SPRED2, thereby inhibiting ERK–NF-κB/STAT3 signaling and restraining tumor growth and metastasis (Yin et al., 2021). In contrast, METTL3 deficiency in myeloid cells reshapes the immunosuppressive TME and diminishes the therapeutic efficacy of PD-1 checkpoint blockade (Xiong et al., 2022). WTAP, in cooperation with YTHDF2, promotes M1 polarization and CD4^+^ T cell infiltration by silencing CES2 mRNA via m^6^A modification, thereby accelerating ulcerative colitis progression. Pharmacological inhibition of WTAP alleviates disease severity, suggesting its potential as a therapeutic target (Xie et al., 2024).

#### 3.2.2 The mechanism of RBPs in the polarization of M2 macrophages

This establishes a positive feedback loop linking SRSF10, glycolytic metabolism, and lactylation. Tumor-derived lactate subsequently promotes M2 macrophage polarization and suppresses CD8^+^ T cell function, shaping an immunosuppressive TME and positioning SRSF10 as a potential biomarker and therapeutic target for immunotherapy resistance. The m^6^A reader YTHDF2 functions as a tumor-intrinsic immune evasion regulator by sustaining tumor glycolysis and suppressing CX3CL1-mediated macrophage recruitment (Yang et al., 2024). YTHDF2 further limits IFN-γ–dependent inflammatory macrophage polarization and antigen presentation, ultimately impairing CD8^+^ T cell activity. Genetic deletion or pharmacological degradation of YTHDF2 remodels the TME toward an immune-activated state and markedly enhances the efficacy of PD-1/PD-L1 blockade (Yang et al., 2024). IGF2BP2 promotes macrophage polarization from an M1 toward an M2 phenotype through an m^6^A-dependent mechanism by targeting TSC1 and activating the STAT6–HMGA2–PPARγ signaling axis. Loss of IGF2BP2 enhances pro-inflammatory M1 polarization and exacerbates inflammation, whereas inhibition of M2 polarization alleviates allergic inflammation, highlighting its therapeutic potential in inflammatory diseases (Wang et al., 2021). Human umbilical cord mesenchymal stem cell–derived exosomes (hucMSC-Exos) modulate macrophage m^6^A landscapes by upregulating METTL3 and YTHDF1, promoting M2 polarization and enhancing Slc37a2 mRNA stability. This suppresses inflammatory macrophage functions and ameliorates inflammatory bowel disease, providing a mechanistic basis for the immunomodulatory effects of hucMSC-Exos. In acute lung injury models, increased expression of GBP4 promotes M1 polarization, while YTHDF1 enhances GBP4 expression by recognizing m^6^A-modified GBP4 mRNA (Xu X. et al., 2024). This regulatory axis controls inflammatory responses and suggests m^6^A modification as a potential therapeutic target in acute lung injury. Multiple tumor contexts further illustrate RBP-driven macrophage polarization. In nasopharyngeal carcinoma, m^6^A-modified WNT2, regulated by ALKBH5 and YTHDF1, stabilizes WNT2 expression (Tang et al., 2025). Secreted WNT2 activates FZD2/β-catenin signaling in macrophages to induce M2 polarization, while simultaneously activating autocrine WNT/β-catenin signaling in tumor cells, forming a positive feedback loop that promotes tumor growth and metastasis (Tang et al., 2025). Similar RBP- and m^6^A-dependent mechanisms regulate macrophage polarization and tumor progression in triple-negative breast cancer, wound healing, abdominal aortic aneurysm, bladder cancer, psoriasis, and gastric cancer liver metastasis, underscoring the broad and context-specific roles of RBPs in macrophage functional reprogramming.

### 3.3 Regulation of macrophage polarization by interactions between RBPs and circRNAs

**FIGURE 3:**
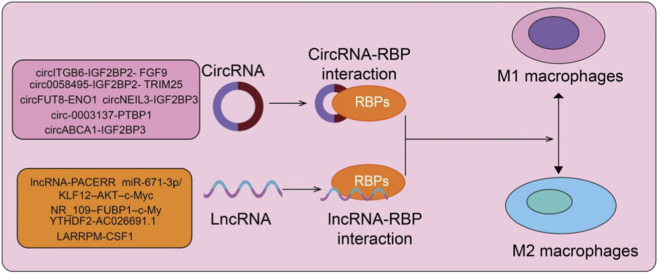
CircRNA and lncRNA interactions with RNA-binding proteins in macrophage polarization. Specific circRNAs (e.g., circITGB6-IGF2BP2, circFUT8-ENO1) and lncRNAs (e.g., PACERR, NR_109) interact with RBPs to modulate macrophage function. These interactions influence the polarization of macrophages toward M1 or M2 phenotypes, thereby regulating immune responses within the tumor microenvironment. Arrows indicate positive regulation, whereas the schematic highlights the molecular crosstalk between noncoding RNAs and RBPs in shaping macrophage-mediated immune outcomes.

Circular RNAs (circRNAs) have emerged as important regulators of macrophage polarization through their interactions with RBPs (Figure 3). In platinum-resistant ovarian cancer, circITGB6 is markedly upregulated and forms RNA–protein complexes with IGF2BP2 and FGF9 mRNA, stabilizing FGF9 expression and inducing M2 polarization of TAMs, thereby conferring cisplatin resistance (Li H. et al., 2022). Targeting circITGB6 blocks M2 polarization and reverses chemoresistance, indicating its potential as a prognostic biomarker and therapeutic target. In pancreatic ductal adenocarcinoma, hsa_circ_0058495 stabilizes IGF2BP2 by preventing TRIM25-mediated ubiquitination and autophagic degradation, thereby activating the MEKK1–ERK pathway to promote tumor proliferation and invasion (Lv et al., 2025). Exosomal transfer of has-circ-0058495 further drives M2 macrophage polarization, reinforcing an immunosuppressive TME. Additional circRNA-RBP axes regulate macrophage polarization across diverse tumor types, including circFUT8-ENO1 in lung cancer (Ren et al., 2025), circ-0003137-PTBP1 in glioblastoma (Liu et al., 2024), circABCA1-IGF2BP3 in clear cell renal cell carcinoma (Ning et al., 2025), and circNEIL3-IGF2BP3 in glioma (Pan et al., 2022). These mechanisms collectively demonstrate how circRNAs function as molecular scaffolds to coordinate RBP activity, metabolic reprogramming, and immune suppression within the TME (Ren et al., 2022).

### 3.4 Regulation of macrophage polarization by interactions between RBPs and lncRNAs

Long noncoding RNAs (lncRNAs) also play pivotal roles in macrophage polarization through direct interactions with RBPs (Rinn and Chang, 2020) (Figure 3). In pancreatic ductal adenocarcinoma, lncRNA-PACERR is highly expressed in TAMs and promotes M2 polarization via the miR-671-3p/KLF12–AKT–c-Myc axis and IGF2BP2-mediated m^6^A-dependent mRNA stabilization (Liu et al., 2022). In the nucleus, lncRNA-PACERR cooperates with KLF12 to recruit EP300 and enhance histone acetylation, forming a positive feedback transcriptional loop that drives an immunosuppressive TME (Liu et al., 2022). Similarly, lncRNA NR_109 is enriched in M2 macrophages and TAMs, where it competitively binds FUBP1 to prevent ubiquitin-mediated degradation, thereby activating c-Myc transcription and promoting IL-4–induced M2 polarization. The NR_109–FUBP1–c-Myc positive feedback loop enhances the pro-tumorigenic functions of TAMs and correlates with poor prognosis in gastric and breast cancers (Zhang et al., 2023). Other lncRNA–RBP interactions, including YTHDF2-mediated degradation of lncRNA AC026691.1 in gastric cancer (Ji et al., 2025), LARRPM-mediated suppression of CSF1 in lung adenocarcinoma (Li Y. et al., 2022), and exosome-transferred LINC01615 in colorectal cancer (Zhang Y. et al., 2022), further highlight the extensive involvement of lncRNA–RBP networks in macrophage polarization and tumor progression. Collectively, these findings underscore the complexity and therapeutic relevance of RBP–ncRNA interactions in shaping macrophage phenotypes and the immune microenvironment. Beyond directing macrophage polarization toward distinct functional states, RBPs also critically influence macrophage survival, stress adaptation, and programmed cell death. Since macrophage persistence or elimination profoundly affects inflammation resolution and tissue remodeling, we next discuss how RBPs regulate key cell death pathways and survival programs in macrophages.

## 4 Regulation of macrophage survival and cell death by RBPs

Macrophage survival and programmed cell death play central roles in the maintenance of tissue homeostasis, regulation of inflammation, and orchestration of immune responses (Bo et al., 2023). RBPs exert fine-tuned control over macrophage fate through post-transcriptional regulatory mechanisms by modulating the expression of mRNAs associated with cell survival, apoptosis, and other forms of programmed cell death (Lu et al., 2020). RBPs can directly interact with mRNAs encoding pro- or anti-apoptotic factors, including members of the Bcl-2 family, caspases, and components of the TNF-α signaling pathway. By regulating the stability and translational efficiency of these transcripts, RBPs enable rapid amplification or attenuation of apoptotic responses in macrophages. For instance, tristetraprolin (TTP) promotes the degradation of TNF-α mRNA, thereby indirectly reducing the accumulation of pro-apoptotic signals and protecting macrophage survival during the resolution phase of inflammation (Zhang et al., 2020). In contrast, HuR (ELAVL1) stabilizes anti-apoptotic mRNAs, enhances cellular stress tolerance, and sustains immune effector functions. Beyond classical apoptosis, macrophages also undergo alternative forms of programmed cell death, including pyroptosis, ferroptosis, and autophagy-associated cell death, all of which contribute to immune regulation (Zhang et al., 2022a). RBPs participate in these processes by controlling the expression of inflammasome-related genes (such as NLRP3 and IL-1β) and mRNAs involved in iron metabolism, thereby influencing the activation threshold and intensity of these cell death pathways. For example, specific RBPs can stabilize or promote the degradation of NLRP3 mRNA, modulating the magnitude of pyroptosis and shaping the scope and severity of inflammatory responses. In colorectal cancer, high expression of RBP-Jκ promotes the secretion of CXCL11, which activates TGF-β1 expression in TAMs, thereby enhancing cancer cell migration, invasion, and EMT (Liu et al., 2021). This positive feedback loop accelerates metastatic progression, and both RBP-Jκ expression and TAM infiltration serve as independent predictors of metastasis and prognosis in colorectal cancer. In sepsis-associated acute lung injury, macrophages release extracellular vesicles enriched in GBP2 and concurrently undergo ferroptosis, which promotes GPX4 ubiquitination and ferroptosis in endothelial cells, ultimately exacerbating lung injury (Li Z. et al., 2025). Targeting the GBP2–OTUD5–GPX4 axis represents a potential therapeutic strategy in this context. In hepatocellular carcinoma (HCC), FOXM1 upregulates IGF2BP3, which stabilizes RRM2 mRNA in an m^6^A-dependent manner, suppressing ferroptosis in tumor cells while promoting malignant behaviors and M2 macrophage polarization (Gao et al., 2025). These findings suggest that targeting the FOXM1/IGF2BP3/RRM2 axis to enhance ferroptosis may provide therapeutic benefit in HCC (Gao et al., 2025). Additionally, lactate has been shown to transcriptionally upregulate IGF2BP2 through H3K18 lactylation (Zhu J. F. et al., 2025). IGF2BP2 binds to and stabilizes Nrf2 mRNA, thereby enhancing ferroptosis resistance in colorectal cancer cells while simultaneously driving M2 macrophage polarization (Zhu J. F. et al., 2025). This dual effect establishes an immunosuppressive tumor microenvironment that promotes tumor growth and metastasis. Collectively, these studies highlight RBPs as critical regulators linking macrophage survival, diverse forms of programmed cell death, and tumor-associated immune modulation.

## 5 RBPs in the regulation of macrophage metabolic reprogramming

Macrophage survival and cell fate decisions are tightly coupled to intracellular metabolic states and energy availability. Accordingly, RBPs-mediated control of RNA metabolism extends beyond cell death regulation to the fine-tuning of metabolic reprogramming, which underlies macrophage activation, functional specialization, and environmental adaptation. Macrophage function is not only governed by signaling pathways and transcription factors but is also highly dependent on cellular metabolic states (Xu R. et al., 2024). The emergence of the concept of immunometabolism has revealed a close interplay between metabolic pathways and immune effector functions (Xu R. et al., 2024). Macrophages dynamically adjust their metabolic programs-such as glycolysis, oxidative phosphorylation (OXPHOS), and lipid metabolism—to support polarization states and functional demands (Xu R. et al., 2024). Pro-inflammatory M1 macrophages exhibit markedly enhanced glycolysis and preferentially engage in aerobic glycolysis (the Warburg effect), even under normoxic conditions, to rapidly generate ATP and metabolic intermediates required for the synthesis of inflammatory mediators and reactive oxygen species (Schwartz et al., 2017). In parallel, the tricarboxylic acid (TCA) cycle is partially disrupted, leading to the accumulation of metabolites such as citrate and succinate, which further reinforce inflammatory signaling and phenotype maintenance (Wu and Minteer, 2019). In contrast, anti-inflammatory and tissue-repair-associated M2 macrophages predominantly rely on oxidative phosphorylation and fatty acid oxidation (FAO) to provide sustained energy supply, supporting anti-inflammatory cytokine production and tissue repair functions (Wu and Minteer, 2019). Enhanced lipid metabolism not only fulfills energetic demands but also contributes to the generation of signaling molecules, such as prostaglandins and lipoxins, which further promote immunosuppressive and reparative responses (Wu and Minteer, 2019). RBPs play a pivotal role in macrophage metabolic reprogramming by regulating the stability and translation of mRNAs encoding metabolic enzymes. Through post-transcriptional control of glycolytic enzymes, mitochondrial respiratory chain components, and lipid metabolism-associated proteins, RBPs influence metabolic pathway selection and indirectly shape macrophage polarization and inflammatory intensity (Sun et al., 2024). This layer of regulation tightly couples metabolic remodeling with functional phenotypes, enabling macrophages to rapidly adapt energy production and immune functions in response to microenvironmental cues (Chen et al., 2025b).

Recent studies have shown that tetrameric pyruvate kinase M2 (PKM2) in macrophages drives glycolytic ATP production, which is subsequently converted extracellularly into adenosine to activate A2a receptors (Palsson-McDermott et al., 2015). This signaling cascade enhances IL-10 secretion and improves mitochondrial function, revealing a PKM2-driven metabolic reprogramming mechanism that promotes anti-inflammatory responses (Palsson-McDermott et al., 2015). In another context, the E3 ubiquitin ligase RNF128 interacts with SRB1 and promotes its Lys63-linked polyubiquitination, enhancing SRB1 membrane recycling and oxidized low-density lipoprotein uptake (Liu Y. et al., 2025). This process induces foam cell formation and inflammatory responses, underscoring the critical role of RNF128-mediated lipid metabolic regulation in atherosclerosis (Liu Y. et al., 2025). Furthermore, in prostate cancer, the histone methyltransferase ASH1L remodels histone methylation patterns in cooperation with HIF-1α, inducing a pro-metastatic transcriptional program in bone metastatic tumor cells (Meng et al., 2025). This reprogramming drives monocyte differentiation toward lipid-associated tumor-associated macrophages (LA-TAMs) and enhances their pro-tumorigenic phenotype, highlighting ASH1L as a key regulator of macrophage metabolic plasticity in bone metastasis and a potential therapeutic target (Meng et al., 2025). In inflammatory arthritis, Pim2 is upregulated in macrophages from patients and mouse models, where it promotes glycolytic reprogramming and M1 polarization by phosphorylating PGK1, PDHA1, and PFKFB2, thereby accelerating disease progression (Xu et al., 2025). Pharmacological inhibition of Pim2 using agents such as HJ-PI01 or neutrophil membrane-coated Bex-PLGA nanoparticles suppresses glycolysis and M1 polarization, alleviating inflammatory arthritis and providing a novel strategy for targeted therapy (Xu et al., 2025). The cumulative effects of RBP-driven regulation of macrophage polarization, survival, and metabolic reprogramming ultimately manifest at the organismal level, contributing to the initiation and progression of diverse diseases. In the final section, we integrate these mechanistic insights to discuss the roles of RBPs in macrophage-associated pathological conditions, including inflammatory disorders, cancer, and metabolic diseases.

## 6 The functions of RBPs in macrophage-related diseases

RBPs play critical roles in the initiation and progression of a wide range of diseases by regulating macrophage polarization, survival, and metabolic states (Huntington and Gray, 2018). Aberrant expression or functional dysregulation of RBPs can disrupt immune homeostasis, leading to imbalanced immune responses that contribute to the pathogenesis of inflammatory diseases, cancer, infectious disorders, and metabolic diseases (Huntington and Gray, 2018). In inflammatory and autoimmune diseases, dysregulated post-transcriptional control of pro-inflammatory and anti-inflammatory cytokine mRNAs by RBPs often results in the persistent activation of chronic inflammation (Huntington and Gray, 2018). For example, loss or functional impairment of tristetraprolin enhances the stability of TNF-α mRNA, thereby driving sustained inflammatory responses (Huntington and Gray, 2018). In contrast, excessive activation of HuR may promote the expression of anti-inflammatory factors, disturbing immune equilibrium. Mechanistically, HuR binds to AU-rich elements in the 3′untranslated regions of target mRNAs, stabilizing transcripts that encode anti-inflammatory cytokines and regulatory molecules. When HuR activity is abnormally elevated, this can lead to an overrepresentation of anti-inflammatory signals, suppressing normal immune activation and impairing pathogen clearance (Huntington and Gray, 2018). Such an imbalance in cytokine production and immune cell activity may contribute to the development of pathological conditions, including autoimmune diseases, where inappropriate immune suppression or dysregulated tolerance disrupts tissue homeostasis. By linking HuR-mediated post-transcriptional regulation to immune equilibrium, this pathway highlights how dysregulation of RBPs can have direct consequences for disease pathogenesis (Huntington and Gray, 2018). Inflammatory cytokines have been shown to induce the expression of RBM43, which suppresses the translation of PGC1α mRNA, leading to reduced mitochondrial biogenesis and oxidative metabolism (Dumesic et al., 2025). This impairment subsequently disrupts energy metabolism and thermogenic capacity in adipocytes. Adipocyte-specific deletion of Rbm43 enhances PGC1α translation, restores oxidative metabolism, improves glucose tolerance, alleviates adipose tissue inflammation, and suppresses cGAS–STING–mediated immune activation (Dumesic et al., 2025). These findings reveal a critical inflammation–RBM43–PGC1α axis in the pathogenesis of obesity-associated metabolic diseases. RBPMS plays a pivotal role in the phenotypic regulation of vascular smooth muscle cells (VSMCs). By binding to myocardin (MYOCD) pre-mRNA, RBPMS modulates alternative splicing to maintain a balanced ratio of MYOCD_v3 to MYOCD_v1 isoforms, thereby promoting contractile differentiation of VSMCs while suppressing fibrotic activity (Imamura et al., 2010). Consequently, RBPMS reduces fibrous cap formation in atherosclerotic plaques and attenuates neointimal hyperplasia following vascular interventions, highlighting its potential therapeutic value in preventing vascular remodeling and restenosis. DRAK2 has emerged as a key regulator in nonalcoholic steatohepatitis (NASH). DRAK2 expression is markedly elevated in the livers of patients and experimental models with nonalcoholic fatty liver disease (NAFLD) and NASH (Li et al., 2021). Mechanistically, DRAK2 interacts with the splicing factor SRSF6 and inhibits its phosphorylation by SRPK1, thereby modulating alternative splicing of genes involved in mitochondrial function (Li et al., 2021). Liver-specific deletion of DRAK2 effectively suppresses the progression from fatty liver to NASH, indicating that DRAK2 represents a potential therapeutic target for NAFLD/NASH (Li et al., 2021). The secreted micropeptide Cf48 promotes renal interstitial fibrosis in chronic kidney disease (CKD). Cf48 is upregulated in renal tubular epithelial cells in CKD, and its serum levels are positively correlated with renal function decline, CKD stage, and the extent of active fibrosis. Cf48 binds to mRNAs of fibrosis-related genes, such as Serpine1, prolonging their mRNA half-lives and enhancing TGF-β1–mediated fibrotic responses, ultimately promoting extracellular matrix deposition (Li et al., 2025b). Genetic deletion or pharmacological inhibition of Cf48 significantly attenuates renal fibrosis in CKD models, suggesting that Cf48 is a promising therapeutic target (Hu et al., 2023).

Ac^4^C is an evolutionarily conserved RNA modification found on tRNAs, rRNAs, and mRNAs, catalyzed primarily by the acetyltransferase NAT10. Recent studies indicate that ac^4^C can enhance RNA stability and translation efficiency (Chen et al., 2023). In macrophages, ac^4^C modification of specific transcripts may fine-tune the expression of genes involved in inflammatory responses and immune signaling. In patients with atherosclerosis (AS), NAT10 expression is elevated and is accompanied by increased levels of ac4C RNA modification. NAT10 regulates macrophage polarization by catalyzing ac4C modification of TLR9 mRNA. Knockdown of NAT10 promotes the transition from M1 to M2 macrophages, suppresses inflammatory responses, and alleviates AS progression, whereas TLR9 overexpression reverses these effects. These findings suggest that the NAT10–ac^4^C–TLR9 axis plays a crucial role in the development and progression of atherosclerosis. Mechanistically, NAT10 catalyzes ac^4^C modification of specific mRNAs in macrophages, enhancing their stability and translation. This modification promotes the expression of TLR9, a key innate immune receptor, thereby skewing macrophages toward a pro-inflammatory (M1-like) phenotype (Yin et al., 2025). The resulting pro-inflammatory macrophages contribute to lipid accumulation, foam cell formation, and vascular inflammation (Yin et al., 2025), all of which drive plaque development and progression. By linking RNA modification to macrophage polarization, this pathway provides a mechanistic explanation for how post-transcriptional regulation can influence atherosclerotic disease progression and highlights potential targets for therapeutic intervention (Yin et al., 2025). Within the tumor microenvironment, TAMs typically exhibit an immunosuppressive M2-like phenotype that supports tumor growth, angiogenesis, and immune evasion. RBPs in TAMs enhance immunosuppressive functions by stabilizing mRNAs encoding VEGF, IL-10, and TGF-β, and by regulating the expression of metabolism-related genes to sustain energy metabolism and phenotypic characteristics. Through these mechanisms, RBPs not only promote tumor progression but also represent potential targets for therapeutic strategies aimed at reprogramming TAMs (Yin et al., 2025). During pathogen infection, RBPs participate in the regulation of macrophage antimicrobial responses by controlling the expression of inflammatory mediators and antimicrobial factors. For instance, HuR enhances the stability of specific interferon-related mRNAs, thereby improving antiviral response efficiency. Meanwhile, the roles of RBPs in metabolic diseases have also gained increasing attention. In obesity and atherosclerosis, RBPs regulate the expression of genes involved in lipid metabolism, influencing macrophage inflammatory activity and foam cell formation, and thereby contributing to disease initiation and progression (Yin et al., 2025). In summary, RBPs exert fine-tuned control over macrophage functions and play indispensable roles across a broad spectrum of diseases. Their dysregulation can lead to excessive inflammation, immune suppression, or metabolic disturbances, positioning RBPs as promising therapeutic targets for the treatment of macrophage-related pathological conditions.

NSUN5 promotes m5C modification of CTNNB1 chromatin-associated RNA, which is oxidized by TET2 and recognized by RBFOX2, leading to β-catenin mRNA degradation and enhanced TAM-mediated phagocytosis of glioma cells (Wu et al., 2024). Targeting NSUN5, alone or combined with IDH1-R132H or CD47/SIRPα inhibition, potentiates TAM activity and suppresses glioma growth *in vivo* (Wu et al., 2024).

## 7 The potential of RBPs as therapeutic targets for immunotherapy

Owing to their central roles in macrophage functional regulation, RBPs have increasingly emerged as promising targets in immunotherapy research. Unlike classical transcription factors, RBPs primarily exert regulatory control at the post-transcriptional level by modulating RNA splicing, stability, subcellular localization, and translational efficiency. This mode of regulation confers RBPs unique and irreplaceable advantages in shaping immune cell fate decisions and functional programs. From a drug development perspective, RBPs often contain well-defined RNA-binding domains or protein–protein interaction interfaces, rendering them more “druggable” than transcription factors and amenable to selective intervention using small molecules or nucleic acid–based therapeutics.

The therapeutic potential of targeting RNA-binding proteins (RBPs) has gained increasing attention in recent years. Several strategies have been explored, including RNA interference approaches such as siRNA or shRNA, antisense oligonucleotides (ASOs), and small-molecule modulators that directly inhibit RBP activity or disrupt their interactions with target RNAs (Cantara et al., 2024). Despite these promising approaches, significant challenges remain for clinical translation, including achieving cell-type-specific delivery, ensuring *in vivo* stability, minimizing off-target effects, and overcoming potential toxicity. Integrating RBP-targeted therapies with existing immunotherapy regimens may further enhance clinical efficacy (Crooke et al., 2021). For example, modulating RBPs that regulate macrophage polarization could improve the tumor immune microenvironment, potentially increasing the responsiveness to immune checkpoint inhibitors or other immune-based therapies (Yu and Yao, 2024). Collectively, these insights highlight both the opportunities and the challenges of RBP-targeted interventions, underscoring their potential as a novel class of immunomodulatory therapeutics.

Importantly, RBPs frequently integrate inflammatory signaling, metabolic reprogramming, and cell survival pathways, thereby functioning as network-level regulators. Targeting RBPs therefore offers the possibility of simultaneously modulating macrophage polarization states, metabolic features, and functional homeostasis through a single intervention, providing novel entry points for immunotherapeutic strategies. Current approaches to targeting RBPs include small-molecule inhibitors, RNA interference technologies (siRNA/shRNA), and antisense oligonucleotides (ASOs), which enable precise regulation of pro- or anti-inflammatory gene expression programs. Notably, RBP-targeted strategies exhibit strong potential for synergistic effects with immune checkpoint blockade therapies, such as PD-1/PD-L1 inhibitors. By reshaping the immunosuppressive phenotype of macrophages within the TME, RBP-directed interventions may amplify antitumor immune responses and offer new combinatorial treatment paradigms for cancer and chronic inflammatory diseases.

For instance, the splicing factor SF3B1 is highly expressed in ovarian cancer and is associated with reduced infiltration of cytotoxic immune cells. Inhibition of SF3B1 induces tumor cell pyroptosis and promotes the release of mitochondrial DNA, which is subsequently engulfed by macrophages, driving their polarization toward a pro-inflammatory M1 phenotype and reshaping an immune-activated TME. Treatment with the SF3B1 inhibitor pladienolide B enhances immune cell infiltration and upregulates PD-L1 expression, thereby synergizing with anti–PD-L1 therapy to elicit potent antitumor effects (Wang S. et al., 2023). The RNA-binding protein IGF2BP2 is highly expressed in the GE-9 epithelial subpopulation of breast cancer and promotes macrophage recruitment via CCL2 signaling, leading to polarization toward M2-like and SPP1^+^ macrophages. This process establishes an immunosuppressive TME and attenuates the efficacy of immunotherapy (Li et al., 2024). Targeting IGF2BP2 effectively remodels the TME, enhances therapeutic responsiveness, and improves clinical outcomes. Radiotherapy (IR) has been shown to upregulate YTHDF2 expression, thereby promoting the expansion of immunosuppressive myeloid-derived suppressor cells (MDSCs), which in turn dampen antitumor immunity and increase radioresistance (Wang L. et al., 2023). Deletion of Ythdf2 in myeloid cells reshapes the MDSC compartment, suppresses their infiltration and immunosuppressive functions, and enhances antitumor immune responses. Mechanistically, IR-induced YTHDF2 expression depends on NF-κB signaling, while YTHDF2 reciprocally activates NF-κB by promoting the degradation of mRNAs encoding negative regulators of NF-κB, forming a positive IR–YTHDF2–NF-κB feedback loop (Wang L. et al., 2023). Inhibition of YTHDF2 alleviates MDSC-mediated immunosuppression and improves the efficacy of radiotherapy and combined radio-immunotherapy. Similarly, SRSF10 enhances tumor glycolysis and lactate production, establishing an SRSF10–glycolysis–H3K18 lactylation (H3K18la) positive feedback loop that promotes M2 macrophage polarization and suppresses CD8^+^ T cell activity, thereby constructing an immunosuppressive TME (Luo et al., 2024). Mechanistically, SRSF10 stabilizes MYB mRNA, upregulating key glycolytic enzymes such as GLUT1, HK1, and LDHA, leading to lactate accumulation. Lactate not only enhances SRSF10 expression through H3K18la in tumor cells but also induces H3K18la in macrophages, activating pro-tumor macrophage functions. Pharmacological inhibition of SRSF10 with the small-molecule inhibitor 1C8 enhances the therapeutic efficacy of anti–PD-1 antibodies, identifying SRSF10 as a potential biomarker and intervention target for immunotherapy resistance.

Strong evidenc showed that, the m^6^A “reader” protein YTHDF2 maintains the pro-tumor phenotype of TAMs by regulating IL-10–STAT3 and IFN-γ–STAT1 signaling pathways (Ma et al., 2023). Loss of YTHDF2 reprograms TAMs toward an antitumor phenotype, enhances antigen presentation capacity, boosts CD8^+^ T cell–mediated antitumor immunity, and suppresses tumor growth (Chen et al., 2025c). Accordingly, TAM-targeted inhibition of YTHDF2 enhances the efficacy of PD-L1 blockade, highlighting its potential as an immunotherapeutic target. In B-cell malignancies, YTHDF2 exhibits dual mRNA reader functions. On one hand, it stabilizes key transcripts through m^5^C–PABPC1 interactions, enhancing ATP production and driving B-cell transformation and tumorigenesis (Chen Z. et al., 2025). On the other hand, YTHDF2 promotes immune evasion via m^6^A-dependent mRNA destabilization. Small-molecule inhibitors targeting YTHDF2 not only suppress tumor growth but also improve the efficacy of CAR-T cell therapy, offering promising strategies for the treatment of B-cell malignancies (Chen Z. et al., 2025). METTL3 enhances the stability of PD-L1 mRNA through m^6^A modification in an IGF2BP3-dependent manner, thereby promoting PD-L1 expression in breast cancer cells. Inhibition of METTL3 or IGF2BP3 reduces PD-L1 expression, enhances T cell activation and infiltration, and strengthens antitumor immune responses, positioning these molecules as potential targets for breast cancer immunotherapy (Wan et al., 2022). NAT10, activated by HOXC8, promotes ac^4^C modification and translation of FOXP1 mRNA, thereby enhancing GLUT4/KHK-mediated glycolysis and increasing lactate secretion (Qin et al., 2024; Li et al., 2025e). This process establishes a lactate-rich TME that facilitates the immunosuppressive activity of regulatory T cells (Tregs). Inhibition of NAT10 significantly enhances the antitumor efficacy of PD-L1 immune checkpoint inhibitors, suggesting its therapeutic potential in cervical cancer immunotherapy. Our recent findings further demonstrate that RBMS1 acts as an immunosuppressive RNA-binding protein in triple-negative breast cancer by stabilizing B4GALT1 mRNA, thereby promoting PD-L1 glycosylation and immune evasion (Zhang et al., 2022c). Targeting RBMS1 enhances T cell–mediated antitumor immunity and improves the efficacy of immune checkpoint blockade and CAR-T therapy. Despite their considerable therapeutic promise, the clinical translation of RBP-targeted strategies faces multiple challenges. First, RBPs often exhibit context-dependent expression patterns and functional diversity across different cell types and tissues, raising concerns about off-target effects and systemic toxicity (Zhang et al., 2022c). Second, RBPs frequently participate in multiple signaling pathways, rendering their functions highly complex and necessitating precise spatiotemporal control. In addition, successful clinical application requires overcoming key challenges related to *in vivo* delivery efficiency, stability, and long-term safety of nucleic acid–based or small-molecule therapeutics. The high functional plasticity of RBPs presents both challenges and opportunities for their development as cancer biomarkers and therapeutic targets. From a biomarker perspective, measurement of total RBP mRNA or protein levels alone is often insufficient to accurately predict patient prognosis. Instead, characterization of specific splice isoforms, post-translational modifications, or subcellular localization may more faithfully reflect functional states. Therapeutically, the dual roles of RBPs increase target selection complexity, as RBPs that promote tumorigenesis in certain cancers may exert protective functions in other tissues or disease stages, and indiscriminate inhibition could result in unintended adverse effects. Moreover, targeting a single pathway may drive RBPs to engage alternative oncogenic networks, leading to therapeutic resistance or tumor recurrence. Therefore, comprehensive dissection of the context-specific determinants governing RBP function-such as specific post-translational modifications or interacting partners-will be essential for developing more selective and personalized therapeutic strategies. Looking ahead, integration of high-throughput drug screening, single-cell omics, and structural biology approaches will enable systematic mapping of RBP-mediated immune regulatory networks, accelerating their translation as immunotherapeutic targets and advancing the realization of precision immunotherapy.

## 8 Conclusion and perspectives

RBPs play fundamental roles in macrophage polarization, survival, programmed cell death, and metabolic reprogramming by orchestrating complex post-transcriptional regulatory networks. These networks not only determine macrophage functional states in inflammation, infection, and tumor microenvironments but also unveil new therapeutic opportunities for immunomodulation. Future studies integrating single-cell technologies, multi-omics analyses, and spatial transcriptomics will be essential for delineating the context-dependent functions of RBPs across diverse tissues and pathological conditions. As an emerging class of immunotherapeutic targets, RBP-directed strategies possess the capacity to modulate multiple signaling pathways and optimize macrophage function, holding great promise for the treatment of cancer and chronic inflammatory diseases and providing a robust theoretical and practical foundation for precision immune interventions.

Recent studies have highlighted the dynamic interplay between tumor cells and the immune microenvironment as a critical determinant of cancer progression and therapeutic response (Gong et al., 2025). In particular, macrophage-driven remodeling of the tumor immune microenvironment has emerged as a key mechanism underlying immunosuppressive states, metabolic adaptation, and resistance to immunotherapy (Gong et al., 2025; Mu and Najafi, 2021). Growing evidence indicates that post-transcriptional regulatory programs, including those orchestrated by RNA-binding proteins (RBPs), are central to shaping macrophage phenotypes and their functional plasticity within the TME, thereby influencing antitumor immunity and treatment outcomes (Gong et al., 2025). From a translational perspective, several practical considerations must be addressed before RBPs can be effectively targeted for therapeutic intervention. Given the pleiotropic and often context-dependent functions of RBPs, achieving sufficient target specificity while minimizing off-target effects and systemic toxicity remains a major challenge. In addition, the feasibility of delivering RBP-targeting agents, particularly in a cell type–or tissue-restricted manner, will be critical for maximizing therapeutic efficacy. Emerging strategies, including RNA-based therapeutics, small-molecule inhibitors, and targeted delivery platforms, may help overcome these limitations. Importantly, rational combination of RBP-targeted approaches with immune checkpoint blockade holds promise for reprogramming the tumor immune microenvironment, enhancing antitumor immunity, and improving patient responses to immunotherapy. Addressing these challenges will be essential for translating mechanistic insights into clinically viable RBP-based therapeutic strategies.
